# Governance, collaboration and community organising in rural Australia: A case study of women’s experiences and contributions to community health and well-being in the Northern Rivers, Australia floods

**DOI:** 10.1177/17455057251345938

**Published:** 2025-06-17

**Authors:** Rebecca McNaught, Emma Pittaway, Loriana Bethune, Dominica Meade, Jo Longman

**Affiliations:** 1 The University of Sydney, Camperdown, NSW, Australia; 2 University Centre for Rural Health, Lismore, NSW, Australia; 3 Griffith University, Southport, QLD, Australia; 4 Gender and Disaster Australia, Melbourne, VIC, Australia; 5 The University of Melbourne, Parkville, VIC, Australia

**Keywords:** floods, community health and well-being, disaster, women’s leadership, community organising, gender, resilience

## Abstract

**Background:**

Whilst gendered violence and other gendered health impacts of disasters are well rehearsed in the literature, relatively less attention has been paid to women’s important role in supporting the health and well-being of communities during the peak and aftermath of extreme weather-related events such as floods.

**Objectives:**

This article explores the gendered dimensions of rural Australian community disaster responses and resilience building, highlighting women’s experiences of and contributions to community health and well-being, disaster governance and leadership in the community setting.

**Design:**

The article presents a case study from the Northern Rivers region of New South Wales, Australia, a rural area on the front line of the climate crisis having been subjected to compounding disasters in the last decade including catastrophic flooding.

**Methods:**

The qualitative study draws upon: semi-structured interviews with community members, government, not for profit and businesses; notes from public events; a research diary and transcripts from a New South Wales Government flood response inquiry.

**Results:**

The results of this study suggest that following the 2022 floods, women have made significant and enduring local contributions to the health, well-being and recovery of communities across the Northern Rivers region. Despite challenges in accessing power and decision-making, women played an essential role in community problem solving and circumnavigated challenges using collaborative local approaches. However, women often paid the cost of undertaking these roles through negative impacts on their own health and well-being.

**Conclusion:**

This study reveals that after disasters, masculinised labour benefits from support and recognition while the prolonged work of women to support the health and well-being of their communities persists without these privileges. Further studies are needed on how women and their leadership strengths can be recognised and supported to enable ongoing health and well-being of disaster-affected communities, including the health and well-being of women organisers themselves.

## Introduction

Climate-related disasters of all kinds, including floods, heatwaves, fires and droughts, have a profound impact on human health.^1^ Climate change is amplifying the intensity, frequency, temporal nature and scale of climate hazards; therefore, the impacts of disasters on human health are growing in magnitude.^2^ These health impacts are not evenly distributed across affected populations. International research across contexts demonstrates that the impacts of disaster are gendered: that is, women, men and people of diverse genders experience disasters differently and unequally.^3–5^ Women also have less economic resilience than men, and they bear the wage-free brunt of caring, emotional support and community recovery in disasters.^6^ Within Australia, recent research on the gendered dimensions of climate-related disasters – including on domestic violence and caring roles – aligns with the international literature.^4,7–9^ These gendered dimensions are often amplified in rural areas.

The gendered social expectations placed on women occur at different scales for example household, community and in the formalised disaster management system and have negative impacts on their health and well-being, as they often place their own mental health and financial needs behind those of their families and communities.^10^ Beyond domestic caring, women often play a critical role in caring for their broader neighbourhood in the aftermath of disasters.^11^ The division of labour in disasters is further reflected in the strongly gendered culture of Australian emergency management agencies. Low numbers of women and people identifying as LGBTQIA+ are evident in paid frontline staff, for example only 10.43% of firefighting frontline staff in New South Wales (NSW) are women.^12^ The disaster response environment has been described as ‘blokey’^13^ and a highly masculinist ‘boys club’ where women experience exclusion, discrimination and barriers to leadership positions.^8–10,14^ A heroic leadership style, often the domain of men, has tended to be celebrated in Australia, while women’s exercising of leadership styles that bring about social change are given less attention.^15^

We recognise that a ‘binary construction of gender’ can be oversimplistic and not reflective of the true nature of the breadth of disaster experiences.^16,17^ However, recent research confirms binary notions are prevalent in the emergency management sector and gender remains a broader socio-structural influence.^16^ We therefore focus our analysis in this article on the experiences of women, noting that individuals experience gender in different ways. In contrast to the large swathe of health literature that focuses on documenting impacts of disasters on women we instead focus this article on women’s contributions to health and well-being of communities post-disaster, using the case study of a catastrophic flood event in NSW.

Global frameworks highlight the importance of understanding both gendered vulnerability to disasters as well as capacities to address them. For instance, the Sendai Framework for Disaster Risk Reduction^18^ highlights that a ‘gender, age, disability and cultural perspective should be integrated in all policies and practices’ (p.13) with women’s participation and leadership needing greater recognition and empowerment. More recently, there have been calls for research examining policies and practices that support gender inclusive disaster management, how women strive for equity in these contexts and the barriers they face in doing so.^19^

Whilst gender is gradually receiving more attention in the Australian disaster literature, it remains under-researched^9,20^ and poorly reflected in emergency management policy. This is despite the launch in 2018 of the National Gender and Emergency Management Guidelines,^21^ Australia’s first national policy on gender and disaster. The nexus of gender, disaster and the impact on individuals’ well-being and the well-being of the community has also not received much attention in Australia. This research addresses a dearth of research on women’s contributions to health and well-being in disaster, particularly in the local informal community organising space.

In this article, we use Hatton’s^22^ ‘mechanisms of invisibility’ framework to analyse and discuss the findings of this research. The three intersecting mechanisms Hatton identifies are: sociocultural (e.g. undervaluing women’s work because they are seen as ‘naturally’ caring), socio-spatial (e.g. undervaluing work performed outside the formal workplace) and sociolegal (e.g. informal labour of community volunteers). These mechanisms intersect to construct the invisibility of women’s volunteer labour in disaster management. Acknowledging the existing work on the influence of hegemonic masculinity in emergency management, and the resulting exclusion of women,^23^ we also apply the notion of hegemonic masculinity to shine light on the gendered dynamics of community-led disaster organising. In the context of high impacts and increasing risks, this article aims to explore the gendered dimensions of disaster resilience at the local level in the Northern Rivers of NSW and how that impacts on the health and well-being of communities. Specifically, the article explores women’s gendered experiences of disaster governance, collaboration and community organising and their contributions to health and well-being of communities in the aftermath of the 2022 floods.

## Methods

### Case study location: The Northern Rivers, NSW

**Figure 1.**
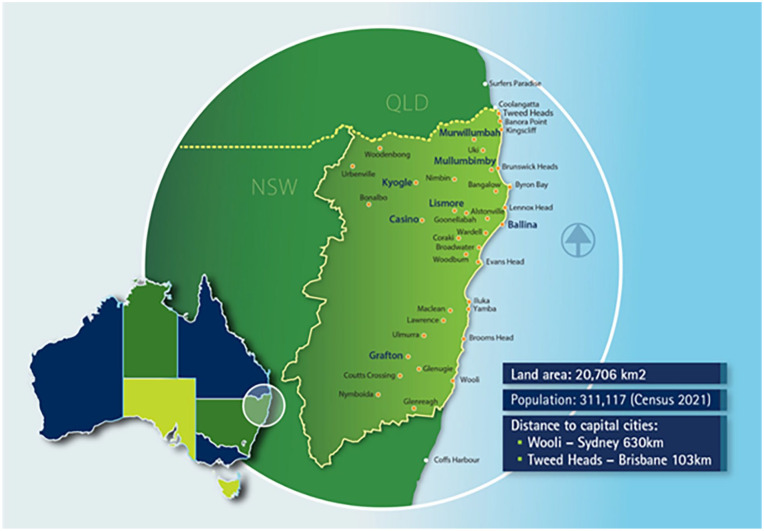
The Northern Rivers region of New South Wales (NSW) on the east coast of Australia is highly disaster prone. Image credit.^30^

**Figure 2.**
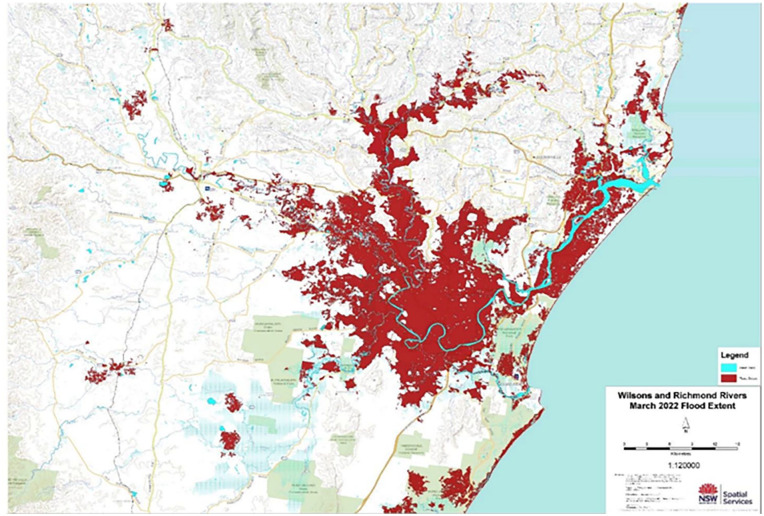
Flood extent map for Wilsons and Richmond Rivers indicating the normal course of the rivers (thin blue line) and the extent of flooding (red area) during the February 2022 flood. Image credit.^31^

The Northern Rivers region of NSW, Australia (see Figure 1) is a highly climate disaster-prone rural region.^24^ In late February 2022 and again in late March 2022, the region experienced catastrophic flooding and landslips. The February flood was the worst in the written history of the region (see Figure 2). Rainfall was exceptional, with the highest weekly total being 1346 mm at Uki, Tweed Shire.^25^ Approximately 11,000 homes were inundated and 13,000 people received support for emergency accommodation from the NSW government.^26^ Primary healthcare facilities were damaged and services disrupted due to staff not being able to access their workplaces.^27^ Emergency services and their communications systems were overwhelmed with the scale of need and many communities were cut off, some for weeks. Affected communities had to, and did, step up in extraordinary ways as disaster ‘victims’ are often the first responders.^28^ The Northern Rivers region has a history of successful collaborative social movements and self-organising which pivoted to provide support across the region.^29^

**Figure 3.**
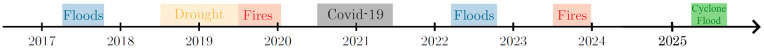
Recent disaster timeline indicating compounding disasters with cascading impacts in the Northern Rivers, New South Wales.

The 2022 floods were not an isolated incident. The Northern Rivers has experienced cumulative disasters in recent years (see Figure 3 for a timeline of recent disasters). Future projections indicate the likelihood of cyclones tracking further south into Northern NSW and the intensification of heavy rainfall events which will in turn increase flooding and landslip risk.^2,24^ Additionally, the confluence of coastal hazards such as sea level rise and storm surges are likely to increase coastal inundation, with four local government areas in the Northern Rivers included in the top 20 most at-risk areas in NSW by 2050.^24^

### Methods for first study

This article draws on two qualitative studies from the Northern Rivers. The ‘Self-organising Systems to Minimise Future Disaster Risk Reduction project (2022–2024)’ was led by the University of Sydney’s Sydney Environment Institute and the University Centre for Rural Health (part of the University of Sydney’s Faculty of Medicine and Health and located in the Northern Rivers). The research focussed on grassroots, informal and unfunded community-led organising during and following the 2022 Northern Rivers floods and landslips, and sought to identify the supports, enablers, barriers and challenges to this type of community response. Detailed findings are available.^32^

The research, within a subtle realist paradigm,^33^ employed a trauma-informed qualitative approach and utilised semi-structured interviews.^34,35^ See Supplemental Material 1 for the study’s interview schedule. Interviews took place between November 2022 and February 2023 and were approximately an hour long, conducted in person or via Zoom. Interviews were followed up by two participant gatherings which provided participants with an opportunity to discuss findings, and to shape them into relevant outputs.

The research team of experienced qualitative researchers was embedded (living and working) in the community prior to the project, and recruitment began with the researchers’ own ‘warm’ networks. Participants were purposively recruited to maximise diversity of: geographic area; size and type of community; type of self-organised group; type of activity undertaken and the role participants played. Ethics approval was obtained from University of Sydney’s Human Research Ethics Committee (HREC approval no. 2022/710).

### Methods for second study

The second study investigated characteristics and outcomes of local level collaboration for addressing climate change and disasters in the Northern Rivers, including collaborative practice between government agencies, private sector and civil society, for example community organisations, universities and non-government organisations. A detailed summary of the ethnographic case study research methods employed in this study has been published.^36^ Data sources including a research diary, notetaking at 27 disaster and climate change themed public events between 2019 and 2023, and 22 semi-structured interviews of around an hour took place with key informants throughout 2021–2022. See Supplemental Material 2 for the study’s interview schedule. In both studies, initial interviews were undertaken to pilot and then refine questions. Additionally, transcripts were utilised from five public hearings of the NSW Government’s ‘Select Committee on the response to the Major Flooding in NSW’ in 2022 which included 26 h of statements from a range of disaster-affected individuals, local politicians (councils, state government representatives), a diverse range of non-government organisations, First Nations groups, businesses, government emergency management personnel and community-based organisations.

Three out of six of the researchers involved in the study were located within the Northern Rivers and were involved in the flood response and recovery efforts in 2022 (in employed and volunteer roles). Rich personal insights were developed playing a close ‘on the ground’ role throughout the event and its aftermath. As per the first study, existing relationships were drawn upon to initially recruit interview participants. These initial contacts were used to snowball recruitment avoiding the pitfalls of cold calling potential interviewees.^37,38^ Research ethics was obtained through Griffith University’s ethics committee (reference number GU 2019/445).

### Data analysis

Interviews in both studies were transcribed (by one of the research team or a professional transcription service), anonymised and stored securely on university databases. All data were uploaded by both study teams into N-Vivo (Version 14 released in 2023 by Lumivero) software.^39^ Qualitative data were analysed thematically in study one, using a combination of codebook and reflexive approaches^33,34^ where an initial codebook (based on research questions and previous literature) was developed and trialled by the research team (including E.P. and J.L.), data deductively coded and the codebook refined throughout multiple team discussions. A more nuanced and inductive approach to capture all data was then pursued and multiple team discussions were held to produce a themes map. Results were coded in the second study using a combination of deductive and inductive coding.^40^ The themes map for study one is provided in Supplemental Material 3, and the codebook for study two has been previously published.^36^ By elaborating on these materials, we have employed a transparent and systematic approach to this research.^41^ The research and interview questions for both projects did not specifically include gender. In both studies, the gendered nature of the community-led disaster organising was a theme developed inductively by the research teams through discussion and reflection during the data analysis process and was flagged as an important area for future research.

### Approach for this article

For the purposes of this article, the datasets from study one and study two (referred to in the results as S1 and S2) were reviewed using a gendered lens. This included extracting data linked with gendered experiences of disaster and climate change governance, gendered contributions to health and well-being of communities and the role of women in local community organising. A gender lens assessing power dynamics and structures was subsequently applied to the selected data, reflecting on the undervaluing of women’s professional skills and roles. The reporting of this study conforms to the Standards for Reporting Qualitative Research (SRQR), with a SRQR checklist provided as Supplemental Material 4.^42^

## Results

The first study included 29 community organisers and volunteers, and in the second study, 22 key stakeholders took part in one-to-one interviews. Transcripts of public events (noted as P) and statements to an Inquiry (W) were reviewed as described above.

### The gendered nature of community disaster organising and care

#### Extent and importance of unpaid community disaster organising led by women

A key finding was the extent and importance of the community-led response to the 2022 floods, which was primarily led by women. Community-led organising encompassed all activities and stages of disaster management (Table 1). Local community members were the first to respond during the floods and landslips, and community-led initiatives remained in place in local communities long after disaster services ended. Community-led response was unique in its ability to draw on local knowledges and understand local needs, particularly among vulnerable and marginalised communities who felt excluded from or mistrustful of mainstream services. This enabled the provision of appropriate, accessible spaces and support. Due to its organic nature, community-led organising responded to community needs with speed and agility. It was often sophisticated and well-organised, drawing on the professional skills and experience of community members. During and after the disaster, community-led organising played an indispensable role in saving lives, providing humanitarian assistance and coordinating disaster recovery and adaptation when emergency assistance and disaster management agencies were absent or inadequate.

This isn’t our day job. Let’s be real here. What we’ve been doing over the past five, six months is the job of the government. And so it’s actually been the community and community groups that have carried this region through the time of crisis. (S2 W2)

**Table 1.** Community-led disaster response activities.

• Boat rescue • Fire-fighting **• Design of systems and databases** **• Running impromptu evacuation centres** • Clean-up **• Managing donations** **• Managing volunteers** **• Cooking and coordinating meals** **• Mental health and well-being support** • Rebuilding	**• Coordinating community recovery hubs** • **Establishing local resilience organisations** • **Grant-seeking** • **Advocacy** • **Participation in formal emergency management structures and recovery processes** • Establishment of Ultra High Frequency (UHF) radio networks • **Establishment of regional networks** **• Animal rescue** **• Case management**

Predominantly women-led activities highlighted in bold although women were involved in all of these activities.

Several participants reflected that women occupied most of the (often unofficial) coordination and leadership roles and undertook the vast majority of the volunteer labour during and after the floods:

I’ve been amazed at how many women have been involved in the response. Yeah, it’s quite amazing when you think of all the – yeah, I mean there’s some blokes around, I’ve got to give them some credit, but, yeah, I’m amazed. . . it was always the women saying, what do you need? What can I help with?. (S1 P1)

Women’s roles in the disaster organising were not therefore limited to unskilled tasks and care-work typically associated with women, such as cooking meals and managing donations. While women performed the majority of this work, they also dominated the leadership and coordination of community-led activities bringing their professional skills and experience to the operations they oversaw. These skills included: event management, IT, nursing, facilitation, communications, community development, clinical psychology, trauma healing, business management, social work and public health.

The gendered nature of this volunteer labour is particularly pronounced in long-term recovery activities. Men’s involvement tended to be concentrated on specific short-term rescue and response activities, while women tended to remain active for months or even years, carrying the responsibility of community recovery and rebuilding. At a regional gathering of grassroots community disaster organisers 2 years after the 2022 disaster, 87% of names on the contact list were female.

The economic burden (and consequent mental health and well-being impact) of this critical contribution to disaster management and community well-being falls to women. Both studies included examples of women taking unpaid time off work to coordinate recovery activities in their local communities because ‘*no one else was going to do it*’ (S1 P3). In the longer term, some women continued to contribute their volunteer labour, while others managed to secure short-term grants and partial funding to continue their recovery work. Even amongst those who secured funding however, the shared feeling was one of overwhelm and exhaustion at the difficulty accessing funding, the necessity of working unpaid hours and their sense of responsibility to their communities which prevented them from stepping back. This contributed to numerous reports of exhaustion and vicarious trauma.

So a lot of what we are doing is dependent on volunteer hours. So I’d like to get paid. I wasn’t expecting to be in this space nearly a year out in an unpaid role. (S1 P16)

#### Contributions to health and well-being of communities by women-led disaster organising

**Figure 4.**
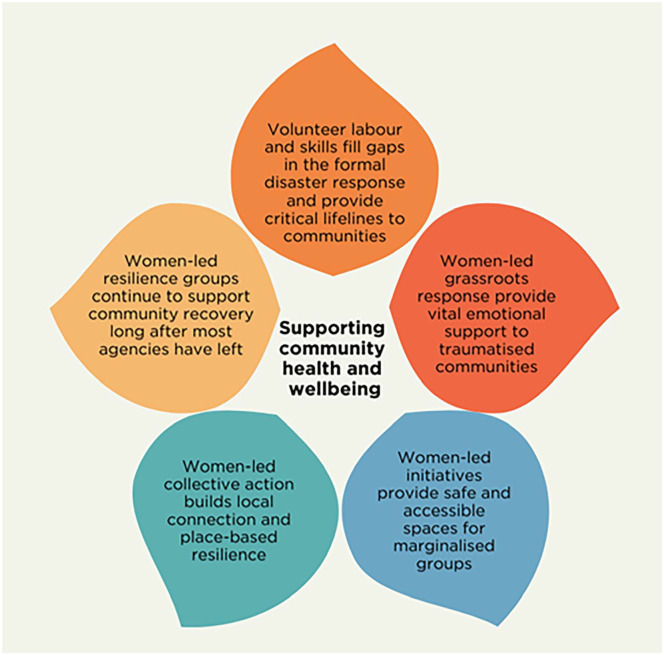
How women-led grassroots disaster response supports community health and well-being.

Both studies illustrated the ways in which community-led disaster response supported community health and well-being (see Figure 4 for a summary). Community members rendered life-saving and basic humanitarian assistance to one another when formal services were absent. This included responding to physical health needs when formal health services were not accessible. This ranged from operating impromptu evacuation centres and feeding people through to medical evacuations and sourcing medications. Apart from conducting rescues and specialised technical skills such as flying helicopters, women performed the majority of this work.

We had the evacuees crying when they were leaving [the impromptu evacuation centre] in gratitude, which was just amazing. They’re calling us the chateau. Because they were just so eternally grateful that they had those first five days just to regroup before they hit the wide world again. (S2 P15)

The community-led response was able to provide emotional support and a therapeutic space in a way that formal services could not. It was usually women who reported stepping into these ‘accidental counsellor’ roles.

Because the community really needed us. And it was more than about food. . . people would just come and then we’d just hug them and they’d just cry. . . And so. . . the food relief turned into something deeper. . . I knew that food was just such an important part of healing. (S1 P29)

The scale of the disaster meant that usual support services disappeared. The community-led response was important in providing this support. For example, some elderly residents were without formal at-home care for months and reliant on fellow community members because the agencies who provided that support were flooded themselves, or because of road closures, or the clients’ flood-damaged houses were deemed unsafe for staff to enter. Formal recovery support services for case management did not become fully operational for many months post-disaster, and again it was mainly women who filled this gap. Community-led organising was also a vital support for marginalised groups, including the queer and First Nations communities, who have a historic mistrust of government agencies and often find mainstream services inappropriate or unsafe.^16,43^

The [First Nations] team helped coordinate food, clothes and essential items for thousands of flood affected residents with no financial support from government for more than three months. (S2 W2)

One participant described the importance of having queer community members on the ground at an evacuation centre to support evacuees who preferred not to interact with government agency staff. They were able to deescalate volatile situations and source gender-affirming and HIV medications. Another woman participant was involved in setting up a First Nations-led culturally safe space for trauma healing.

### Women’s invisibility and exclusion from disaster governance

#### Women’s needs and participation in formal disaster governance lacking

During the flood response and in the subsequent recovery, there was a tension between two distinct cultures: the culture of top-down emergency management agencies (which are highly gendered spaces) and the ‘bottom-up’ culture of community-led recovery (dominated by women). Participants expressed a need for more gender-neutral conditions for women to feel safe to participate in disaster governance, including the presence of women in the room, meetings being chaired to proactively support women’s participation and include discussion of the needs of women and children.

It was all the boys club. . .I remember sitting in one meeting early on. . . and every face in the meeting was a white middle-aged guy with a buzz cut. And, and I was like, there is no women. There is no diversity. There was no sense of community or that whole recovery space. (S2 P19)

Women emphasised a feeling of being unheard, dismissed and their perspectives not being valued or listened to.

I just think that sometimes, you know that they [men] seem to be perceived, and listened to more than a woman doing the same thing. (S2 P10)

#### Lack of recognition of the importance of community organising led by women

Lack of recognition of the role played by community-led response was also repeatedly identified as a key challenge. During the disaster, this meant that emergency management agencies ignored or refused to share information and resources with community-led operations, even when the community had been on the ground organising the response for hours, days or weeks prior to the arrival of formal agencies. It was also evident in dismissive attitudes displayed by some emergency management personnel.

Helicopters and things started to come over and police on trail bikes started to roll in. None of them – we didn’t know that any of that was happening, none of them stopped at the [community-led recovery] hub to talk to us or anything like that. (S1 P2)

During the recovery, lack of recognition was evident in various ways. Some community-led grassroots groups were excluded from funding opportunities despite them being central to the recovery in many local areas. They were also excluded from disaster management communications and decision-making. This included not being invited to participate in discussions about recovery planning and changes to emergency management procedures.

It was really interesting because they were putting together a community response action group, something like this, or a committee. And there was no members of the community there! (S1 P22)

The gendered nature of community-led organising appeared to contribute to its invisibility and allow some disaster management personnel to dismiss and devalue it. In some instances, women were patronised for their assumed lack of expertise which was to the detriment of the safety of communities downstream to them:

We started reporting to the State Emergency Services and to council our concerns of [the water moving through the catchment] that was about to hit [town] and they were all sort of excused. ‘Sweet lady’ Council says, ‘Oh you always ring this in when the causeway is over’. I said, ‘I’m really serious. I’m like, in my 28 years, I’ve never seen it like this’. (S2 P9)

In another case, the heroic actions of community members who took part in flood rescue were celebrated by a local council, without any equivalent celebration of the women who had collectively contributed many thousands of unpaid hours towards the recovery effort:

The experience of prioritising in the messaging of like, ‘oh, these men in their dinghies, what heroes!’ . . . But here we are with just simply a trillion women doing all of the childcare, all of the cooking, all of the soft labour, literally everything plus being on dinghies. . . and there’s just nothing for us. The sustainability of recovery is such a feminised, deprioritised aspect. (S1 P15)

This lack of recognition negatively impacted the health and well-being of communities by undermining the critical work being undertaken by community-led initiatives. Community-led recovery hubs went without access to vital resources such as radios, petrol and tools in the early days, and in the longer term, some were forced to close due to a lack of funding. The dismissal of local community knowledge also meant emergency services missed out on vital intelligence, jeopardising their own response to impacted communities.

#### Creative circumnavigation through collaboration

Feeling unheard and needs remaining unmet, along with the catastrophic scale of the disaster, were the catalysts for innovative, participatory and collaborative responses from women. These included women organising motorbike riders to take local doctors to locations inaccessible by helicopter, and women working collaboratively together to establish and run evacuation centres:

For me, when I got down to that evacuation centre, I just expected that someone would be there in charge. I was expecting that it would be functioning and the reality was that it wasn’t and it took individuals who were willing to just step up and say, ‘Well, I’m here and I can see there’s a need for this,’ and doing it. (S1 P14)

In response to a lack of involvement in the formal decision-making, women often brought together what they described as ‘unholy alliances’ in order to garner support and be responsive to needs on the ground. These ‘unholy alliances’ included siloed government departments, local politicians, farmers, environmentalists and lawyers being brought together to improve outcomes for communities and reverse the sometimes negative impacts of top-down state-level decision-making. Examples included a lack of clear communication, consultation and risk management in the government’s implementation of housing buy-backs and when choosing locations for temporary accommodation, which impacted on people’s mental health and on the physical health of children.

One participant described herself as being the ‘mortar between the bricks’ to enable change, finding joint language and vision across organisations beyond the language of disasters to assist communities to thrive. Participants described women’s leadership style as ‘inclusive’, ‘empathetic’ and ‘conversational’.

You just have to look at who was there at the community centre, who was taking the charge, that was leading it, it was an all women operation. And it was lovely, I think there’s a level of empathy and a level of just getting in there and getting your hands dirty and making things happen that women have, in that sort of a role, more so than men. (S2 P17)

## Discussion

### Women’s contribution is more than ‘baking cakes’

The results of this study suggest that following the 2022 floods, women made significant and enduring contributions to the health, well-being and recovery of local communities across the Northern Rivers by undertaking roles of emotional and practical support, filling gaps in disaster management responses, providing safe spaces for flood-affected populations, and promoting connection and belonging in their communities. However, study participants described paying the price of undertaking these roles through negative impacts on their own health and well-being. These community organising roles challenge the extent of gendered division of labour often written about^4,6,7^ because women’s contribution is not limited to and defined by caring roles and an extension of domestic labour, but is characterised by high levels of professionalism and expertise. Many women reported using their extensive professional skills and networks to undertake well-organised and complex disaster management operations. They often described using their awareness of gaps in the formal response and recovery and the context-specific needs of their communities to draw together coalitions of support to enable change. Through creative circumnavigation and collaborative practice, deficits were overcome. Existing literature does not yet elaborate well on the leadership roles of women in post-disaster recovery settings.

Over 2.5 years post-flood, this is still occurring. Despite formal response and recovery systems that stand up and stand down, larger NGO programmes that come and go, the authors have observed that many in-situ women are still working on efforts to repair, connect, recover and prepare their communities. This is evidenced in the results of this article by the proportion of female names represented in the Northern Rivers Community Resilience Alliance. Government disaster support focuses disproportionately on short-term recovery, meaning that community-led initiatives may be the only help available to community members still struggling after the funding and services have ended. At the other end of the spectrum, this research suggests that ongoing women-led collective action builds stronger community connection and capacity, in turn contributing to increased resilience to future adversity. Our findings support the limited previous research indicating the important role and leadership styles that women play in community connection and mutual support in disaster recovery.^6,11,44^

### The invisibility of women’s labour

Despite the extensive and sophisticated unpaid disaster recovery efforts led by women, participants of this study emphasised that their work was often dismissed as unimportant and that the significant contribution of women remains largely unrecognised. A gender lens has been absent in formal reports (e.g.^45^). Government inquiries into the 2022 disaster have failed to acknowledge the value of community-led disaster organising or its gendered and long-term nature.^31,46^ Women’s labour in Australia is historically undervalued, with professions dominated by women such as healthcare and education sectors receiving lower earnings compared to male dominated industries.^47^ These findings correlate with previous disaster-related literature which see men’s disaster relief efforts emphasised in the media.^28,48^ The lack of funding to support women’s community-level efforts also lies in stark contrast to large increases to the budgets of government emergency services since the floods. For example, the NSW State Emergency Service received an additional $88.3 million in 2023 as outlined in their annual report.^49^

Applying Hatton’s^22^ threefold framework (socio-cultural, socio-spatial and socio-legal as defined in the introduction), we can identify the intersecting mechanisms by which women’s informal work relating to community health and well-being in disaster management is rendered invisible. The socio-cultural construction of gender roles means that the caring and community-building aspects of community response and recovery are essentialised as expressions of women’s ‘natural’ caring qualities and are thus not recognised as ‘work’.^22^ Our research suggests that socio-cultural expectations on women to provide prolonged unpaid labour means that women disproportionately shoulder the burden of rebuilding their communities following a disaster. The invisibility of women’s labour then creates a positive feedback loop: because women do the majority of post-disaster community organising, it is automatically less visible as work. In addition, the skilled labour and professional expertise women contribute is subject to a double layer of invisibility because of the prevalent assumption that community responses tend be chaotic and disorganised.^50^

In terms of socio-spatial invisibility,^22^ much community-led disaster organising is performed in spatially ambiguous ways. Disaster-affected communities and grassroots groups often do not have appropriate premises available for their operations, meaning work is undertaken in people’s homes or in makeshift structures or village halls, sometimes filling the gap caused by absent emergency and government services – who therefore do not ‘see’ the work being undertaken. Socio-legally,^22^ women’s position as informal volunteers contributes further to their invisibility because they fall outside the legal definitions of employment, and their organisations are emergent and often not yet incorporated. The lack of recognition of informal labour as a gendered phenomenon contributes not only to its invisibility but also to women’s structural exclusion from formal emergency management roles.

### The influence of hegemonic masculinity

Our results suggest that spatial, cultural and legal mechanisms that determine the visibility of work played out very differently in the case of the male-dominated flood rescues in the Northern Rivers. Participants of our study pointed out the recognition and support provided on both an institutional and community level for actions such as boat rescues carried out mainly by men (the so-called ‘tinny army’) in comparison to the invisible work of women after the disaster, further reflecting the gendered nature of community recognition. This study reveals how after disasters, gender influences the visibility of informal work. Heroism is valorised, while the ‘mortar between the bricks’ role that women often play in collective action is often unrecognised and invisible.^28^ This demonstrates the influence of hegemonic masculinity: the legitimation of men’s dominance over women through the promotion of a socially constructed masculine ideal.^51^

The role of hegemonic masculinity in contributing to a hypermasculine emergency management culture at the exclusion of women from emergency management roles is already recognised.^23,52^ This study sheds light on how it also influences the informal community response, shaping the activities men are involved in and the visibility and status given to ‘masculine’ roles. Men’s labour benefits from support and recognition while the prolonged work of women persists without these privileges. Hegemonic masculinity not only excludes women from formal emergency management spaces but our study suggests that it also renders invisible the informal work women do alongside and in the absence of disaster management agencies. Women’s work and their inclusive and empathic leadership in communities, as described by participants in this study, actively resists the male-model style of leadership that favours patriarchal ways of organising.^53^ Our study suggests that the influence of hegemonic masculinity had implications for the ways in which the Northern Rivers community valued and recognised leadership during the disaster recovery.

This study finds that the chronic devaluation of women’s contributions to the health and well-being of the Northern Rivers region has significant implications. Results suggest that a lack of consideration of women’s post-disaster support to communities undermines women’s efforts and has a negative impact on the community groups they lead, who struggle to maintain adequate funding to survive. The invisibility and outright dismissal of knowledge of community-based approaches and needs is in stark contrast to international humanitarian settings, where gender, inclusion and protection are identified as issues to take seriously within disaster response decisions.^54,55^ Lack of consideration of gender, inclusion and protection in disaster decision-making threatens the health and well-being of the communities women support and of the women themselves, which supports other studies undertaken in the region post-flood.^56^ The implication of this research is that the invisible labour dominated by women must be acknowledged and acted upon in order to improve health outcomes for disaster-affected communities.

### Where to from here

NSW disaster management policies clearly articulate a need for ‘shared responsibility’ between government, private sector, NGOs and communities.^57^ At present, our research indicates that community efforts and by nature of the Northern Rivers example, women, are taking on risk and responsibility without adequate support. This correlates with findings of recent research drawing upon cases across NSW, Australia: ‘formal disaster recovery processes routinely overlook, leave out or treat as an add-on the participation and leadership of impacted communities and consultation with them’ (p.2).^52^ State plans also often emphasise the importance of emergency management agencies building awareness of risks in communities, rather than actively co-designing plans that value and incorporate local knowledge and actions.^36,57^ Investment in community leadership and coordination platforms are important features of community development, enabling cohesion and building skills within impacted communities.^58^ Our research demonstrates that this recognition and involvement would improve the mental health outcomes for community (women) leaders as well as assist communities to have their broader health needs addressed in a post-disaster environment.

Through enhanced efforts to link communities to the formal emergency management sector and disaster resilience governance and increasing access to funding for community groups, opportunities for women to participate in decisions will be increased. Decision-making also has increased chances of being informed of needs ‘on the ground’ when community organisers are better connected to and have a say within formal structures (including gendered needs). Women are more likely to shoulder unpaid caring responsibilities in communities, including single-headed families and caring for family members with disabilities.^6^ Providing avenues for these perspectives to be heard is important for tailoring disaster response and recovery to the health needs that they observe. Gendered reporting in disasters is also important for ensuring diverse health needs are addressed and contributions acknowledged.^21^

Although providing ‘a seat at the table’ can be important, it is not just ‘adding women and stirring’.^44^ The table and notions of leadership may have to be redesigned through different imaginings of what it means to be a leader. As noted by Damousi and Tomsic^15^: ‘women’s leadership should be more than just adding women in, but be more reforming, and shifting public images and imagination about what good leadership is and how it can be executed’ (p.4). The strengths and attributes of women’s community leadership, such as co-leading and collaborative approaches, are to be celebrated. Recognising affected communities beyond a ‘victim’ narrative is linked with psychological well-being and recovery.^28^ Without targeted efforts to support this work from institutions and government, women-led organising will continue to go unacknowledged and unvalued and health of communities and women in particular, not optimally supported.

### Limitations

We have chosen to focus on women’s roles in this article; however, we acknowledge that the experiences and roles of the queer community in community organising was also immense. Research focussed specifically on these perspectives would assist move the literature towards a less binary understanding of disaster agency and highlight the important contributions of the queer community to health and well-being. Additionally, the gendered findings in this article were a theme that emerged organically from the data of the two studies and gender was not the main focus in either study. The results presented in this study are observations from participants which arose spontaneously during their reflections of their role and work in community-led response efforts. Had we asked more deliberate gendered questions we may have received more in depth and nuanced responses which could have added to the material and reflections shared here. A gender-specific study could investigate the implications of informal community organising for the wider voluntary landscape in disaster-affected communities. The Northern Rivers case of the 2022 flood disaster was particularly catastrophic, and the findings might not be the same in other locations. Further studies in Australia are worth undertaking to ascertain similarities and differences of findings in different geographies.

## Conclusion

Previous research has focussed on exploring women’s increased domestic caring roles post-disaster, and the health impacts of disasters on women. In contrast, this article investigated women’s contributions to health and well-being via community-led disaster organising. We found that following the 2022 Northern Rivers NSW floods, women made significant and enduring contributions to the health, well-being and recovery of local communities. This included providing emotional and food-related support, filling gaps in disaster management responses, providing safe spaces for flood-affected populations, and promoting connection and belonging in their communities. Despite the extensive and sophisticated unpaid disaster recovery efforts led by women, their experience was that their work in the informal sphere remains largely invisible and unrecognised. This article found that notions of hegemonic masculinity where male roles are celebrated, extended beyond the realm of formal emergency management, into the informal community responses to disasters in Australia. The strengths and attributes of women’s community leadership, such as co-leading, collaborative, ‘mortar between the bricks’ approaches, need to be acknowledged for the critical role they play in post-disaster recovery of communities. As the climate becomes more hostile, large-scale disasters become more likely, and these scenarios need to be prepared for. Our research points to the need for further deliberate studies investigating the agency of women in disaster recovery and preparedness. More specifically, how these efforts can be captured and valued and how women can be supported in these roles to enable not only the ongoing health and well-being of disaster-affected communities, but also of women organisers themselves.

## Supplemental Material

sj-docx-1-whe-10.1177_17455057251345938 – Supplemental material for Governance, collaboration and community organising in rural Australia: A case study of women’s experiences and contributions to community health and well-being in the Northern Rivers, Australia floods Supplemental material, sj-docx-1-whe-10.1177_17455057251345938 for Governance, collaboration and community organising in rural Australia: A case study of women’s experiences and contributions to community health and well-being in the Northern Rivers, Australia floods by Rebecca McNaught, Emma Pittaway, Loriana Bethune, Dominica Meade and Jo Longman in Women's Health
