# Supplementary material for: Governance, collaboration and community organising in rural Australia: A case study of women’s experiences and contributions to community health and well-being in the Northern Rivers, Australia floods
Source: Womens Health (Lond). 2025 Jun 17;21:17455057251345938. doi: 10.1177/17455057251345938 (PMC12174688; doi:10.1177/17455057251345938)
Supplement: sj-docx-1-whe-10.1177_17455057251345938 – Supplemental material for Governance, collaboration and community organising in rural Australia: A case study of women’s experiences and contributions to community health and well-being in the Northern Rivers, Australia floods [file sj-docx-1-whe-10.1177_17455057251345938.docx]

**Supplementary Material**

1. **Interview Schedule Study 1**
2. Tell us a little bit about your connection to the Northern Rivers/Hawkesbury/Blue Mountains area. How long you have been in the area?
3. What communities do you feel a part of or belong to?
4. How were you involved in community disaster response and/or recovery efforts?
5. Why did you become involved? Is there anything in your background or experience that you feel equipped you for this involvement?
6. Did you work with others in your efforts? Who and in what ways?
7. What worked well and what would you do differently?
8. Do you feel there are strengths of community-led responses compared to more formalised responses? If so, what are they?
9. How do you think government agencies could support communities to better prepare for and respond to future disasters?
10. What did you learn from your experience that you would like other communities to know? What supported you to keep going?
11. **Interview Schedule Study 2**
12. Can you please tell me about your current role – what are your responsibilities and key areas of work?
13. How do climate change and disasters intersect with your work?
14. How are these issues addressed in the organisation that you work in?
15. What words come to mind when you think of collaboration?
16. Please think of a specific local level collaboration you’ve been involved with that addressed vulnerability to climate change and disasters
17. Starting conditions – who initiated the collaboration? What motivated different types of actors to work in a collaborative process?
18. Design- How was the collaboration designed? What type of actors where involved in designing it? How would you describe the arrangement – formal/informal?
19. Process-What did the collaboration involve? Can you outline the steps that were taken as a collective? What challenges were encountered during the process?
20. Outcomes - What were the outcomes of the process itself? They could be positive and/or negative, large or small.
21. System: how did broader systemic factors interact with the collaborative arrangement?
22. Did partners have a positive or negative history of working together before they started the collaboration? Did conflict/disagreement occur during the process and if so, how was it addressed?
23. Did some organisations/individuals have more power over making decisions than others? How did this play out?
24. What skills do you think are important for facilitating successful collaboration?
25. What does collaborative leadership mean to you?
26. **Themes Map Study 1**


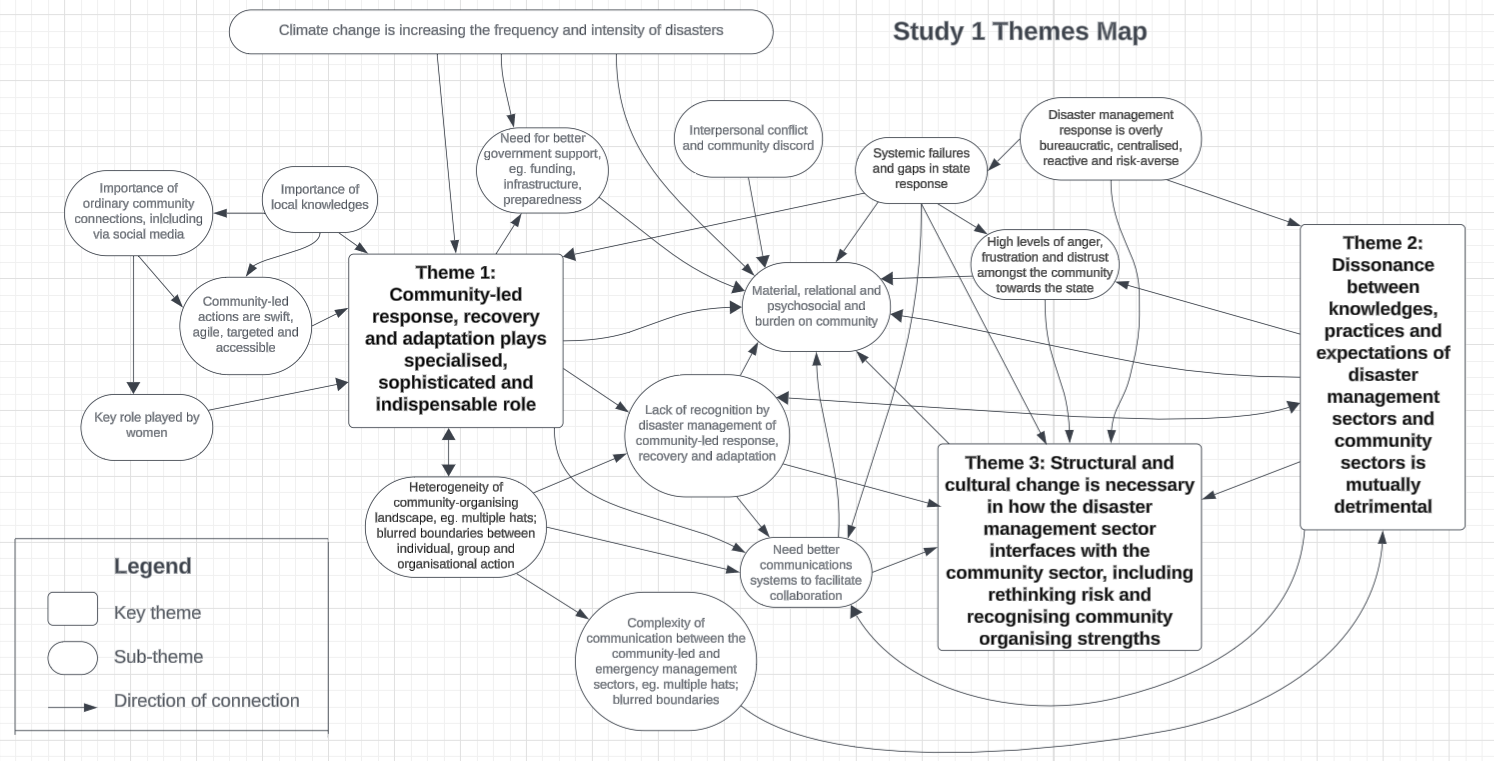


1. **SRQR Checklist (1)**

| **No.** | **Topic** | **Description of how criteria is addressed in the paper** | **Page** |
| --- | --- | --- | --- |
| S1 | Title | **Description of the nature of the topic** – Title: includes “case study” (which incorporates numerous methods) | **1** |
| S2 | Abstract | **Summary of key elements of the study including sub-titles -**  Abstract: background, objectives, design, methods, results, conclusion | **1** |
| S3 | Problem formulation | **Description and significance of the problem/phenomenon studied, review of relevant theory and empirical work; problem statement** - Introduction: climate change, gendered dimensions of disasters, lack of literature on women’s contributions to community health and wellbeing in disaster recovery, Hatton’s invisibility framework, hegemonic masculinity | **2-4** |
| S4 | Purpose or research question | **Purpose of the study and specific objectives or questions** – Introduction: In the context of high impacts and increasing risks this paper aims to explore the gendered dimensions of disaster resilience at the local level in the Northern Rivers of NSW and how that impacts on the health and wellbeing of communities | **3** |
| S5 | Qualitative approach and research paradigm | **Qualitative approaches outlined** – Methods: within a subtle realist paradigm for the first study and including case study with ethnographic dimensions for the second study. Guiding feminist theory outlined in introduction. | **5** |
| S6 | Researcher characteristics and reflexivity | Methods: A detailed prior description of the ethnographic research methods employed in the second study has been published previously and is referenced. Personal attributes of authors outlined, including unique insights from some of the authors living within the case study area throughout the 2022 flood event. | **6** |
| S7 | Context | Methods: Elaboration on the Northern Rivers case study location, including rationale based on compounding disasters and catastrophic flooding event in 2022. | **4-5** |
| S8 | Sampling strategy | Methods: Method for sampling outlined, including snowball and purposive sampling techniques. | **5-6** |
| S9 | Ethical issues pertaining to human subjects | Methods: Human research ethics approval references included for both studies. | **5-6** |
| S10 | Data collection | Methods: Types of data collected outlined (i.e. semi-structured interviews, flood inquiry hearing transcripts, notes from public events, research diary), start and stop dates of data collection and analysis included. | **5-6** |
| S11 | Data collection instruments and technologies | Methods: We note that our research questions did not specifically include gender (the focus of this paper), but the theme of gender emerged inductively through a process of discussion and reflection during the data analysis. | **5-6** |
| S12 | Units of study | Results: Number and relevant characteristics of participants, documents, and events included in the study. | **7** |
| S13 | Data processing | Methods: Methods for processing data prior to and during analysis, including transcription, use of NVIVO software, codebook development, and anonymization/deidentification of excerpts. | **5-6** |
| S14 | Data analysis | Methods: Qualitative data were analysed thematically, using a combination of codebook and more reflexive approaches. Methods employed in the second study have been published previously and a reference provided. | **6** |
| S15 | Techniques to enhance trustworthiness | **Techniques to enhance trustworthiness and credibility of data analysis -** Methods: for study 1 a detailed description of the process of data analysis is included and two references provided which guided this process. Methods employed in the second study have been published previously and a reference to this is provided. | **6** |
| S16 | Synthesis and interpretation | Results and Discussion: Main findings integrated with prior research or theory (Hatton’s invisibility framework, hegemonic masculinity and broader disaster literature) | **7-15** |
| S17 | Links to empirical data | Results: Evidence (quotes) substantiate analytic findings | **7-11** |
| S18 | Integration with prior work, implications, transferability, and contributions to the field | Discussion: Short summary of main findings appear in conclusion; discussion explains how findings and conclusions connect to, support, elaborate on earlier scholarship; application of the implications of the findings to broader settings discussed; identification of unique contribution to scholarship which is to identify and make visible women’s contributions to community health and wellbeing in disaster recovery in an Australian context. | **12-16** |
| S19 | Limitations | Discussion: Trustworthiness and limitations of findings discussed in dedicated section of discussion | **15** |
| S20 | Conflicts of interest | Declared | **16** |
| S21 | Funding | Sources of funding and other support declared | **16** |

1. O’Brien BC, Harris IB, Beckman TJ, Reed DA, Cook DA. Standards for Reporting Qualitative Research: A Synthesis of Recommendations. Academic Medicine. 2014;89(9):1245-51.
